# In Silico Prediction of the Phosphorylation of NS3 as an Essential Mechanism for Dengue Virus Replication and the Antiviral Activity of Quercetin

**DOI:** 10.3390/biology10101067

**Published:** 2021-10-19

**Authors:** Lamya Alomair, Fahad Almsned, Aman Ullah, Mohsin S. Jafri

**Affiliations:** 1 Department of Biostatistics and Bioinformatics–Bioinformatics Section, King Abdullah International Medical Research Center, Riyadh 11481, Saudi Arabia; OmairL@NGHA.MED.SA; 2 The School of Systems Biology and the Krasnow Institute for Advanced Study, George Mason University, Fairfax, Manassas, VA 22030, USA; falmsned@masonlive.gmu.edu (F.A.); aullah3@gmu.edu (A.U.); 3 King Fahad Specialist Hospital—Dammam, Dammam 32253, Saudi Arabia; 4 Center for Biomedical Engineering and Technology, University of Maryland School of Medicine, Baltimore, MD 21201, USA

**Keywords:** dengue, NS3, phosphorylation, quercetin

## Abstract

**Simple Summary:**

Dengue is a mosquito-borne virus that infects up to 400 million people worldwide annually. Dengue infection triggers high fever, severe body aches, rash, low platelet count, and could lead to Dengue hemorrhagic fever (DHF) in some cases. There is currently no cure, nor a broadly effective vaccine. The interaction of two viral proteins, nonstructural Proteins 3 and 5 (NS3 and NS5), is required for viral replication in the infected host’s cells. Our computational modeling of NS3 suggested that phosphorylation of a serine residue at position 137 of NS3 by a specific c-Jun N-terminal kinase (JNK) enhances viral replication by increasing the interaction of NS3 and NS5 through structural changes in amino acid residues 49–95. Experimental studies have shown that inhibition of JNK prevents viral replication and have suggested that the plants’ flavonoid Quercetin, Agathis flavone, and Myricetin inhibit Dengue infection. Our molecular simulations revealed that Quercetin binds NS3 and obstructs serine 137 phosphorylation, which may decrease viral replication. This work offers a molecular mechanism that can be used for anti-Dengue drug development.

**Abstract:**

Dengue virus infection is a global health problem for which there have been challenges to obtaining a cure. Current vaccines and anti-viral drugs can only be narrowly applied in ongoing clinical trials. We employed computational methods based on structure-function relationships between human host kinases and viral nonstructural protein 3 (NS3) to understand viral replication inhibitors’ therapeutic effect. Phosphorylation at each of the two most evolutionarily conserved sites of NS3, serine 137 and threonine 189, compared to the unphosphorylated state were studied with molecular dynamics and docking simulations. The simulations suggested that phosphorylation at serine 137 caused a more remarkable structural change than phosphorylation at threonine 189, specifically located at amino acid residues 49–95. Docking studies supported the idea that phosphorylation at serine 137 increased the binding affinity between NS3 and nonstructural Protein 5 (NS5), whereas phosphorylation at threonine 189 decreased it. The interaction between NS3 and NS5 is essential for viral replication. Docking studies with the antiviral plant flavonoid Quercetin with NS3 indicated that Quercetin physically occluded the serine 137 phosphorylation site. Taken together, these findings suggested a specific site and mechanism by which Quercetin inhibits dengue and possible other flaviviruses.

## 1. Introduction

Dengue virus (DENV), also known as break-bone fever, is a global health concern afflicting around 400 million individuals in more than 100 countries [1,2]. DENV causes severe illness, and sometimes, a potentially deadly complication called Dengue hemorrhagic fever (DHF) [3,4]. The prevalence of Dengue fever has grown dramatically worldwide in recent decades. Dengue’s global spread poses a severe health threat because there are neither specific drugs to treat nor a broadly effective vaccine to prevent Dengue infection [5,6,7,8,9,10,11,12]. Recent studies indicated that the plant flavonoids Quercetin, Agathisflavone, and Myricetin inhibit Dengue infection and bind to the same specific site of the viral nonstructural protein 3 (NS3) [13,14]. However, the exact mechanism for this inhibition remains unclear. In this study, we performed a bioinformatic analysis of the Dengue proteome to identify amino acid modifications by kinases that are likely to affect viral interaction partners in the human host. We then used molecular simulation to test the functional consequences of phosphorylation at specific sites in NS3. Finally, we studied the predicted binding site for the antiviral drug Quercetin to increase understanding of its mode of action.

**Figure A1:**
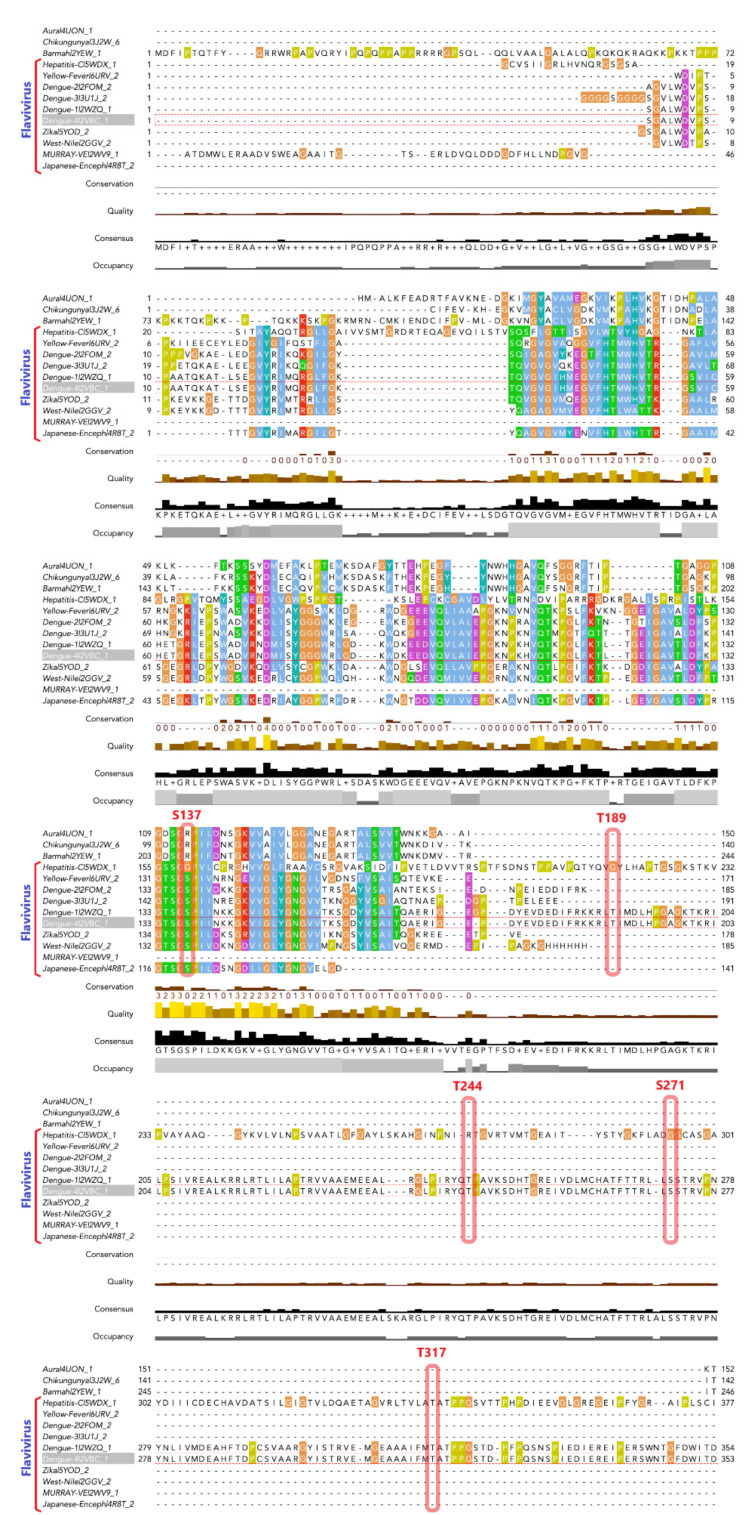

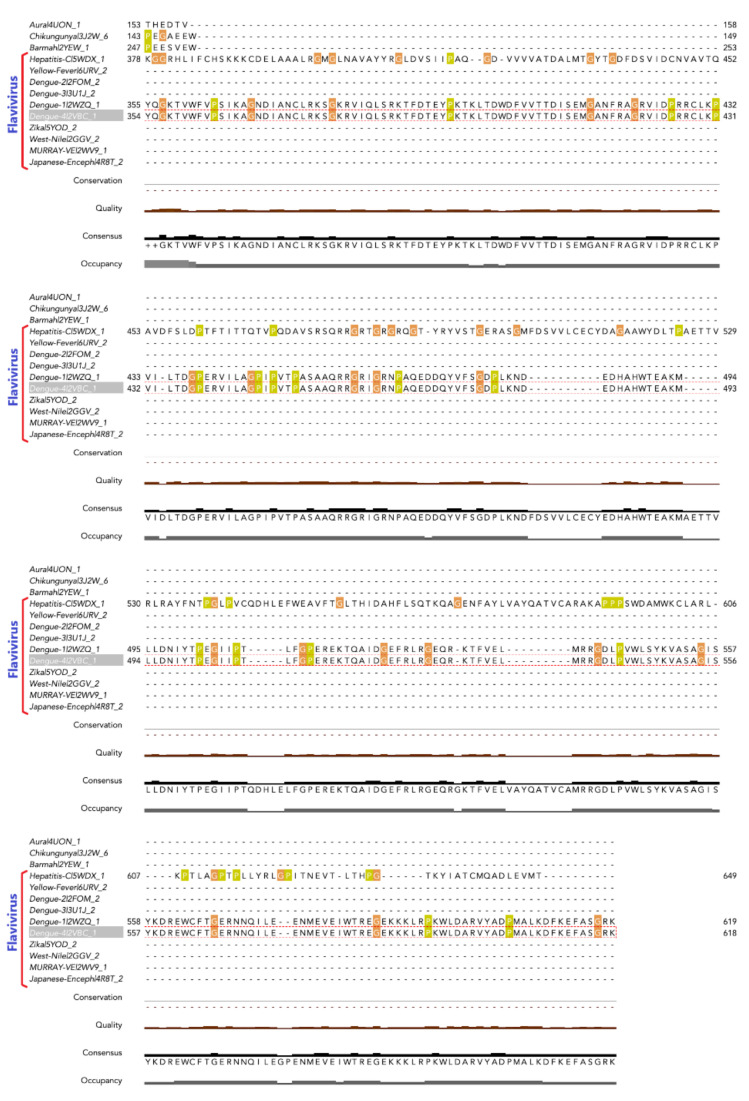
Multiple Sequence Alignment of *Flavivirus* Family. Dengue viruses (all four serotypes), hepatitis C virus, yellow fever virus, zika virus, West Nile virus, Murray Valley encephalitis, and Japanese encephalitis are all members of the *Flavivirus* family. Aura virus, Chikungunya virus, and Barmah virus (carried by mosquitoes but not of the *Flavivirus* family) are included for comparison. The *Flavivirus* family shows a high homology degree, especially in the regions flanking S137 indicated by the red box and text. The degree of homology is less in the other proposed phosphorylation sites T180, S244, S271, and T 317.

**Figure A2:**
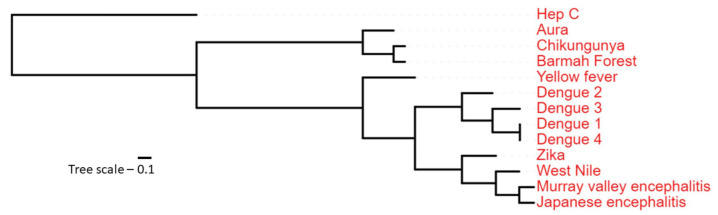
Phylogenetic Tree for *Flavivirus* Family. Most of the *Flavivirus* family viruses show a close relation, with hepatitis C diverging from the rest. The non-*Flavivirus* family viruses cluster separately.

The DENV genome encodes a polyprotein of 3391 amino acid residues with a gene order of 5′-C-prM-E-NS1-NS2A-NS2B-NS3-NS4A-NS4B-NS5-3′, ten viral proteins, including three structural proteins, and seven nonstructural [15]. The structural proteins are responsible for virion formation. In contrast, the nonstructural proteins play roles in the synthesis of viral RNA replication [16]. The viral proteins such as nonstructural protein 3 (NS3) and nonstructural protein 5 (NS5) that take part in viral replication and viral protein synthesis enter the host cell and migrate to the endoplasmic reticulum (ER) membrane, which is the site of protein synthesis in the host cell, to use cellular pathways for viral replication [17,18,19]. DENV is a member of the Flaviviridae family that includes the West Nile virus (WNV), yellow fever virus, Zika virus, and Hepatitis C virus (HCV). NS3 shares a high degree of homology with other members of this family, as shown in the amino acid multiple sequence alignment in Figure A1 found in Appendix A [20,21,22,23,24]. The relationship of members in this family based on this alignment is shown in Figure A2.

Both NS3 and NS5 are viral proteins that are vital components in DENV replication. Additionally, NS3 and NS5 contain conserved motifs found in several RNA helicases and RNA-dependent RNA polymerases, respectively [25]. Phosphorylation of NS5 is critical to its function and association with NS3. Prior research has supported a mechanism where the phosphorylation state of NS5 controls the association and disassociation of NS3 with NS5, which affects viral replication [26]. NS3 is the second-largest key component in DENV replication machinery. The multifunctional enzyme NS3 performs various viral replication actions and plays an essential role in antiviral evasion [27]. However, there is a gap in our understanding of the role of NS3 in molecular mechanisms underlying the replication of DENV.

Our lab recently used a computational approach, which predicted that NS3 could be phosphorylated by around 500 human kinases [28]. We hypothesized that inhibition of kinases responsible for phosphorylation might inhibit viral replication. We predicted the kinases that are most likely to phosphorylate NS3 by using neural networks and other machine learning algorithms to calculate and rank the score of top kinases that phosphorylate DENV NS3 [28]. We applied a range of computational methods, including molecular simulations, to classify the functional impact or phosphorylation of NS3 structure on viral replication at the molecular level. This paper explores the structural effects caused by NS3 amino acid residue phosphorylation at the two sites, serine 137 (S137) and threonine 189 (T189), and the potential impact of these structural effects on NS3 and NS5 interaction, and, consequently, on DENV viral replication. These two proteins were chosen for study because NS3 and NS5 are considered to be promising drug targets [22]. Lastly, we predicted a mechanism behind the anti-viral Quercetin action through its interaction with NS3.

## 2. Materials and Methods

The molecular dynamics trajectories and the supporting files are available as Supplemental Material in the Mason Archival Repository Service (MARS): https://doi.org/10.13021/dwk3-ys70 (accessed on 13 October 2021.)

### 2.1. Predicting Phosphorylation

To investigate possible amino acids that might be phosphorylated by human kinases, we analyzed the NS3 amino acid sequence by three online tools: GPS 3.0 (http://gps.biocuckoo.org/online.php, accessed on 10 July 2019), NetPhos 3.1 (http://www.cbs.dtu.dk/services/NetPhos/, accessed on 10 July 2019), and Scansite 3 (https://scansite4.mit.edu/, accessed on 10 July 2019). These tools were chosen because a recent study rated them as among the most reliable phospho-algorithms [29,30,31,32]. The candidates were ranked using a combination of the highest scores and a site being found by all three tools using a prediction score cutoff (GPS > 9, NetPhos > 0.4, and Scansite > 0.4). The 19 top candidate sites (listed in Table A1) were further filtered to the 5 sites that were identified by all three algorithms. These 5 sites were compared using the SASA scores of < 0.1 were retained. The remaining 3 candidates were compared by multiple sequence alignment (MSA) across the NS3 dengue virus sequences for sequence conservation. The most conserved predicted phosphorylated sites (S137 and T189, in bold) were chosen for further study by molecular simulation. Multiple sequence alignment was carried out using Clustal Omega (V 1.2.4) under the default setting and visualized using Jalview (V 2.11.1.3) [33,34].

### 2.2. System Preparation for Molecular Simulation

#### 2.2.1. Initial Structure

**Figure 1:**
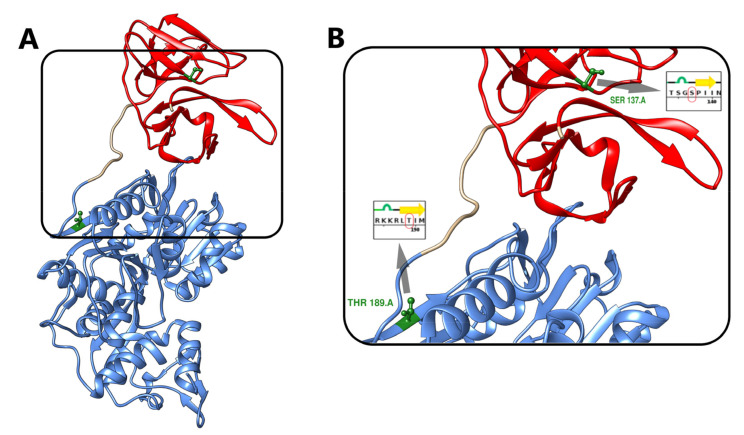
NS3 crystal structure. (**A**) The crystal structure of the NS3 Protease-Helicase from Dengue virus (PDB: 2VBC). The Protease domain (N-terminal, residues 20–168) is shown in red. The linker (169–179 is uncolored. The Helicase domain (C-terminal, NS3Hel, residues 180–618) is shown in blue. (**B**) Serine 137 residue (S137) and Threonine 189 residue (T189) location in NS3 conformation and its position in the amino acid sequence (inset-circled in red).

We retrieved the initial NS3 crystal structure (PDB: 2VBC) from the Protein Data Bank (PDB) [35,36] (Figure 1A). The initial structure has been visualized and processed using UCSF Chimera (v 1.14). Structures were viewed using Visual Molecular Dynamics viewer (VMD v1.9.3) [37].

#### 2.2.2. Phosphorylated Structure

Since NS3 phosphorylation at serine 137 and threonine 189 was our study’s focus, we used the Visual Molecular Dynamics viewer (VMD, v1.9.3) to generate two NS3 phosphorylated systems; S137 and T189. S137 was generated using SP2 phosphoserine patch and T189 using the THP2 phosphothreonine patch. The phosphorylation patches were saved in the Supplementary Materials in the Mason Archival Repository Service (MARS repository at https://hdl.handle.net/1920/11628 (accessed on 13 October 2021)) as “psfgen_phosphorlation137.pgn” and “psfgen_phosphorlation189.pgn”.

#### 2.2.3. Solvation

The three NS3 structures (the unphosphorylated NS3 (WT), S137, and T189) were solvated in a cubic periodic box with a three-site transferrable intermolecular potential (TIP3P) water model with minimum distance from the protein surface using VMD [38]. The wild-type system had 36,163 atoms (9517 molecular atoms and 26,646 water molecules). Each phosphorylated protein system had 36,166 atoms (9520 molecular atoms and 26,646 water molecules).

#### 2.2.4. Simulations Steps

The Molecular Dynamics (MD) simulation was prepared and run by Nanoscale Molecular Dynamics (NAMD 2.13 for Win64-multicore-CUDA) with the CHARMM36 all-force field parameters parallel programming model [39,40]. Periodic boundary conditions were applied, and structures were reported every picosecond (ps). We used a 12 Å cutoff for Van Der Waals interaction with a switching function distance of 8 Å, and the smooth particle-mesh Ewald (PME) method was enabled accordingly. Before the MD simulation, all the flowing procedures were applied to all three systems.

#### 2.2.5. Minimization

Each system was energetically minimized to relax possible steric clashes and obtain a low energy start conformation. A total of 2000 steps of minimization were performed. We used the NAMD script “2VBC_wb_min.namd”, which is included as Supplementary Materials in the MARS repository, to perform this step.

#### 2.2.6. Heating

Each system was heated from 273 K to the average physiological temperature (300 K) for 300 ps. The Langevin thermostat was applied. The temperature was incremented slowly by 0.001 K. We used the NAMD script “2VBC_wb_heat.namd”, which is included as Supplementary Materials in the MARS repository, to perform this step.

#### 2.2.7. Equilibration

Each system was equilibrated to adjust the system density under Isothermal–isobaric (NPT) ensemble conditions for 200 ps. We used the NAMD script “2VBC_wb_equil.namd”, which is included as Supplementary Materials in the MARS repository, to perform this step.

#### 2.2.8. MD Simulation

Each system was then MD simulated to sample the structural characteristics and dynamics at 300 K using the microcanonical ensemble (NVE) for 100 nanoseconds (ns) under Isothermal–isobaric (NPT) ensemble and with a time step of 1 femtosecond (fs). The long-range electrostatics was handled using the particle-mesh Ewald (PME) method [41]. The atom coordinates were recorded every 1 ps throughout the simulation. Moreover, 1 fs integration step was used for all simulations. We used the NAMD script “2VBC_wb_quench.namd”, which is included as Supplementary Materials in the MARS repository, to perform this step.

#### 2.2.9. Trajectories Analysis

The root means square deviation (RMSD), root mean square fluctuation (RMSF), and principal component analysis (PCA) were calculated for all trajectory structures using the Bio3D R package (v 2.4-1) [42,43]. Hierarchical cluster analysis of the scaled Ψ and Φ was conducted using stats R package (v 3.6.2) (https://www.rdocumentation.org/packages/stats/versions/3.6.2, accessed on 1 January 2020). The RMSD, RMSF, Ramachandran (Ψ and Φ angles), PCA, and hierarchical clustering plots were generated using the ggplot2 R package (v 3.3.2) (https://ggplot2.tidyverse.org/authors.html, accessed on 1 January 2020) and the ggpubr R package (v 0.4.0) (https://cran.r-project.org/web/packages/ggpubr/index.html, accessed on 1 January 2021). We used the dplyr (v 1.0.2) (https://cran.r-project.org/web/packages/dplyr/index.html, accessed on 1 January 2021) and the tidyverse R package (v 1.3.0) (https://cran.r-project.org/web/packages/dplyr/index.html, accessed on 1 January 2021) for data manipulation. The analysis of covariance (ANCOVA) was used to analyze the significance between RMSD means for the three simulated structures (unphosphorylated WT at time 100 ns, S137 at time 100 ns, and T189 at time 100 ns) while correcting for the within- structures time steps variability using the rstatix R package (v 0.6.0) (https://cran.r-project.org/web/packages/rstatix/index.html, accessed on 1 January 2020). Post hoc pairwise comparisons of estimated marginal means (adjusted means) were performed to identify which pairs are different using the emmeans R package (v 1.5.3) (https://cran.r-project.org/web/packages/emmeans/index.html, accessed on 1 January 2020). The Bonferroni multiple testing correction is applied [44].

#### 2.2.10. Docking

The 3D structures of interacting proteins can provide valuable atomic-level information regarding the protein-protein interface details. We used ClusPro 2.0, a protein-protein docking algorithm server, to evaluate the docking of WT, S137, and T189 against NS5. [45,46,47]. The MD simulation frame was used for each of the three dockings at time 100 ns atomic coordinates against NS5 (PDB: 2J7U). The docking of Quercetin (PubChem CID: 5280343) was performed against the protease domain of NS3 (PDB: 2VBC) using Autodock Vina (v 2.0) [48]. The Python scripts in the PyRx Virtual Screening software (v 0.9.8) were used to analyze the docking results [49]. UCSF Chimera (v 1.14) provided for interactive visualization and analysis of molecular structures [50].

#### 2.2.11. Binding Pocket Calculation

The Computer Atlas of Surface Topography of Proteins (CASTp) was used to identify the location and calculate the volume of the protease domain solvent housing pockets. The radius cutoff was set to 1.4 Å [51].

## 3. Results

In silico studies combined phosphorylation site prediction algorithms (GPS 3.0, NetPhos 3.1, and Scansite 3) followed by MD simulations were applied to assess the proteins (WT, S137, and T189) physical behavior [52,53]. Lastly, we used protein–protein docking to evaluate phosphorylation’s effect on the docking against NS5.

### 3.1. Phosphorylation Sites Prediction

The investigation of possible protein phosphorylation sites revealed many potential candidate positions on NS3 and several corresponding human kinases by all three algorithms (GPS 3.0, NetPhos 3.1, and Scansite 3), along with their score and residue position in the sequence. Identifying the top kinases required a three-tiered approach. In the first tier, we used the three phosphorylation site prediction tools, which suggested a total of 1489, 953, and 108 possible phosphorylation sites with the associated phosphorylating kinases, respectively. In many cases, a particular site was predicted to be phosphorylated by multiple kinases. In the second tier, we considered kinases that have a combination of a high score in one of the three algorithms. The number of kinases decreased to 61, 76, and 64, respectively. In the third tier, we took the top 19 sites from each algorithm in the second tier (Table A1). Next, only the sites that were identified in all three algorithms were retained. The phosphorylation of Ser and Thr sites found on the surface likely deactivate function, while those toward the interior likely activate function as has been suggested based on the argument that phosphorylation developed later in evolution [54]. For this reason, only the 3 sites with SASA scores <0.1 were retained. Evolutionarily, phosphoproteins are subject to more sequence conservation than their non-phosphorylated counterparts [55,56]. Hence, a multiple sequence alignment (MSA) using Jalview (www.jalview.org, accessed on 1 January 2020) was applied to identify the most conserved regions among NS3 dengue virus sequences [34].

The two most evolutionarily conserved candidate sites on NS3 are serine 137 (S137), which was predicted to be phosphorylated by MAPK, GSK3, CDK1, or, and the residue threonine 189 (T189), which was predicted to be phosphorylated by Kinase AKT, PKB (AKT), or CAMK2G. NS3 contains an N-terminal protease domain (residues 19–168) and a C-terminal (residues 180–618) helicase domain and linker (residue 169–179) (Figure 1). Both domains have been reported to have enzymatic activity and to be involved in NS3–NS5 interaction [57,58]. We examined the structural effects of phosphorylating one amino acid residue from each domain to gain insight into phosphorylation’s role in both domains. A graphical representation of DENV NS3 (PDB: 2VBC) and the two sites (S137 and T189) is presented in Figure 1. The domain (N-terminal) of the NS3 (residues 19–168) is shown in red, the linker (residues 169–179), and the Helicase Domain (C-terminal) of NS3 (residues 180–618) are shown in blue. In Figure 1B, the S137 residue (main panel and circled in red in inset), can be found in the N-terminal domain. The T189 residue (shown in blue in the main panel and in cyan in the inset) can be found in the C-terminal domain.

### 3.2. Effects of NS3 Phosphorylation and Conformational Change in Protein Structure

We examined the three NS3 structures (WT, S137, and T189) behavior under normal physiological conditions using MD simulation. The three simulations were performed for 100 ns at 300 K. Periodic boundary conditions were used with structures reported every 1 ps to study the phosphorylation’s structural effects on NS3 structures.

**Figure 2:**
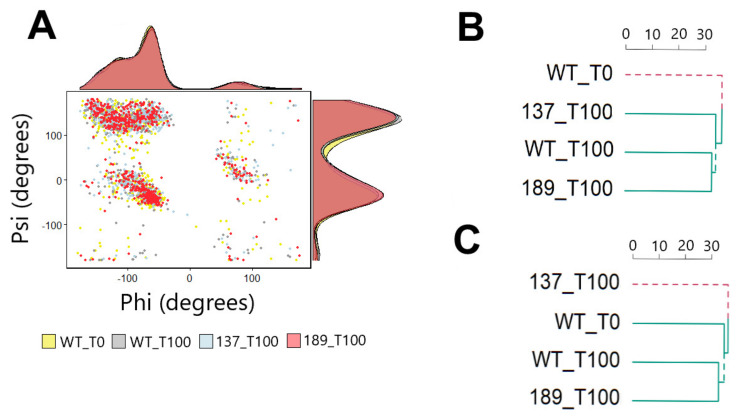
Ramachandran plot of NS3 backbone angles. (**A**) The Ramachandran and density plots for all Ψ/Φ backbone angles for unphosphorylated WT at time 0 (yellow), unphosphorylated WT at time 100 ns (gray), S137 at time 100 ns (light blue), and T189 at time 100 ns (light red). (**B**) Hierarchical clustering plot of all Ψ angles (**C**) Hierarchical clustering plot of all Φ angles.

Figure 2 shows a Ramachandran plot and marginal density plot of all Ψ/Φ angles in four PDB structures: (1) unphosphorylated WT at time 0 ns, (2) unphosphorylated WT at time 100 ns, (3) S137 at time 100 ns, (4) T189 at time 100 ns (Figure 2A). When using all Ψ/Φ angles, the four structures show a close pattern suggesting overall structure preservation with no significant conformational change than the original protein (unphosphorylated WT at time 0). The hierarchical clustering results for all Ψ angles showed the simulated structures (unphosphorylated WT at time 100 ns, S137 at time 100 ns, and T189 at time 100 ns) had a tendency to cluster together (Figure 2B). Interestingly, the hierarchical clustering results for all Φ angles showed the tendency of unphosphorylated WT at time 0, unphosphorylated WT at time 100 ns, and T189 at time 100 ns to cluster together that suggested a significant conformational change in S137 at time 100 ns (Figure 2C).

**Figure 3:**
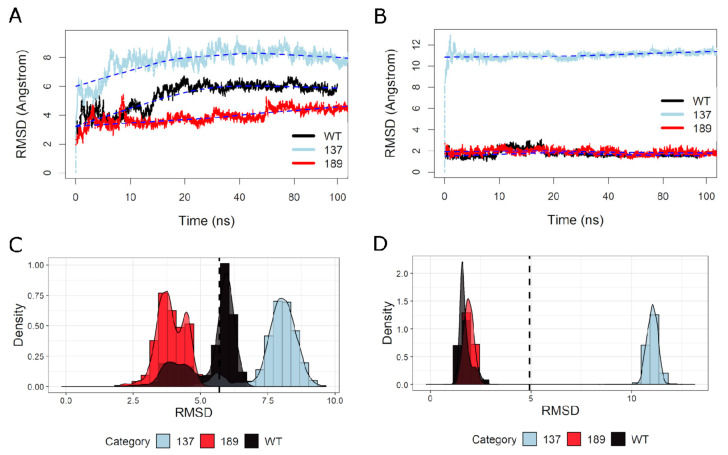
Root mean square deviation (RMSD) vs. MD simulation time and RMSD density of the three simulated NS3 structures. (**A**) The RMSD of three simulated NS3 “all” amino acid residues throughout the MD simulation compared to the starting structure (unphosphorylated WT at time 0 ns). The WT is shown in “black,” S137 is in “light blue,” and T189 is in “red.” (**B**) The RMSD density plots of each of three simulated NS3 “all” amino acid residues throughout the MD simulation compared to the starting structure (unphosphorylated WT at time 0 ns). The mean of all RMSD values = 5.6 is shown by the black vertical line. (**C**) The RMSD of three simulated NS3 amino acid residues (49–94) throughout the MD simulation compared to the starting structure (unphosphorylated WT at time 0 ns). (**D**) The RMSD density plots of each of three simulated NS3 amino acid residues (49–94) throughout the MD simulation compared to the starting structure (unphosphorylated WT at time 0 ns). The mean of all RMSD values = 5 is shown by the black vertical line.

We tracked the RMSD of all residues for the three MD simulated structures (unphosphorylated WT at time 100 ns, S137 at time 100 ns, and T189 at time 100 ns) throughout the simulation compared to the starting confirmation (unphosphorylated WT at time 0 ns) to measure the effect of phosphorylation of serine 137 and threonine 189 on the NS3 protein behavior (Figure 3). The residues simulation showed a clear separation between the three simulated structures at time 100 ns (Figure 3A). S137 had the highest RMSD, followed by WT. T189 had the lowest RMSD compared to the other two simulated structures. The same pattern is reflected in the three simulated structures’ RMSD density plots (Figure 3B). After adjustment for the simulation time steps, there was a statistically significant difference in all restudies RMSD between the groups, F(2, 9590) = 59,671.861, *p* < 0.0001 (Table 1). We repeated the RMSD tracking for NS3 residues 49 to 94 only. S137 had the highest RMSD to both T189 and WT, which showed very close RMSD fluctuations throughout the simulation (Figure 3C). RMSD density plots showed a clear separation of S137 from T189 and WT (Figure 3D). After adjustment for the simulation time steps, there was a statistically significant difference in The (49–94) residues RMSD between the groups F(2, 9590) = 1,296,396, *p* < 0.0001 (Table 2). The RMSD score was statistically significantly greater in S137 (11.002 ± 0.0045) compared to T189 (1.908 ± 0.0045) and WT (1.733 ± 0.0045), *p* < 0.001 (Table 3).

The RMSD differences between the unphosphorylated (WT) and the phosphorylated proteins (S137 and T180) for the functional and structural domains of the NS3 protein are shown in Table 4. This includes the N-lobe (residues 20–168), the linker (169–179), the C-lobe (180–618), the catalytic domain, and the NS3–NS5 interaction domain (66–85). Phosphorylation of NS3 at S137 caused a larger change in RMSD of the N-lobe catalytic domain compared to the WT. A large change in the N-lobe was also observed with phosphorylation at T189. The changes in the C-lobe are small, with the changes in residues 566–585 only slightly larger. The linker region also shows significant change.

**Figure 4:**
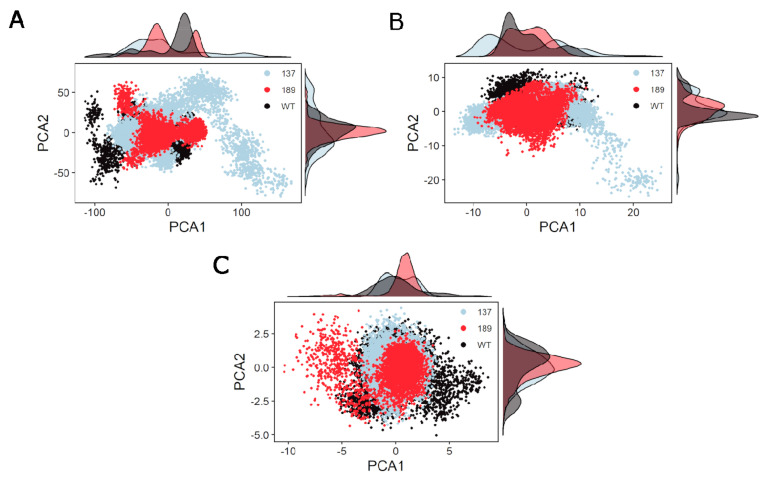
Principal component analysis (PCA) of the cartesian coordinates through the simulation for the three simulated NS3 structures. (**A**) Principal component 1 vs. principal component 2 of three simulated NS3 “all” amino acid residues throughout the MD simulation compared to the starting structure (unphosphorylated WT at time 0 ns). The WT is shown in “black,” S137 is in “light blue,” and T189 is in “red.” (**B**) Principal component 1 vs. principal component 2 of three simulated NS3 amino acid residues (49–94) throughout the MD simulation compared to the starting structure (unphosphorylated WT at time 0 ns). The WT is shown in “black,” S137 is in “light blue,” and T189 is in “red.” (**C**) Principal component 1 vs. principal component 2 of three simulated NS3 amino acid residues (566–585) throughout the MD simulation compared to the starting structure (unphosphorylated WT at time 0 ns). The WT is shown in “black,” S137 is in “light blue,” and T189 is in “red.”

To further explore the difference between NS3 in the unphosphorylated state (WT) and phosphorylated at residue S137 or residue T189, a principal component analysis was performed on the cartesian coordinates of the amino acid residues (Figure 4). There are significant differences between the three structures in PC1 (Figure 4A). NS3 phosphorylated at S137 is different from unphosphorylated (WT) and phosphorylated at T189 in PC1 for residues 49–94 (Figure 4B), Table 5. Figure 4C shows that the differences in 566–585 between the three states display more overlap in the distributions.

### 3.3. Protein Movement Changes with Phosphorylation

**Figure 5:**
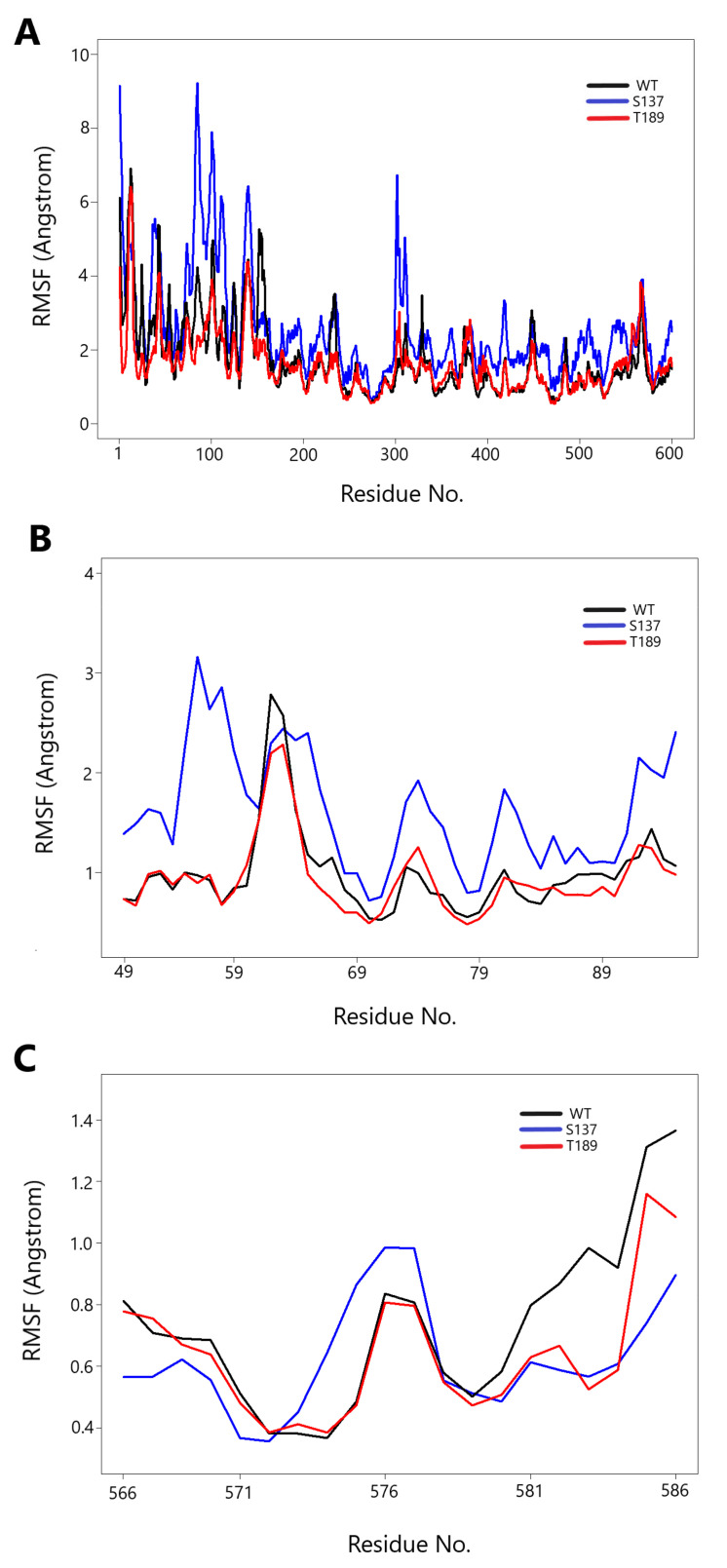
Root mean square fluctuation (RMSF) vs. MD simulation for the three simulated NS3 structures. Phosphorylation at serine 137 (S137) increases NS3 protein all residues fluctuation. (**A**) The RMSF of three simulated NS3 “all” amino acid residues throughout the MD simulation compared to the starting structure (unphosphorylated WT at time 0 ns). The WT is shown in “black,” S137 is in “blue,” and T189 is in “red”. (**B**) The RMSF of three simulated NS3 (residues 49–95) shows an evident rise in RMSF value in S137 residues (40–50) compared to WT and T189. (**C**) The RMSF of three simulated NS3 (residues 566–586) shows a close pattern of the three simulated structures.

The RMSF measures the mobility of the protein backbone amino acid residues. We calculated RMSF for three MD simulated structures (unphosphorylated WT at time 100 ns, S137 at time 100 ns, and T189 at time 100 ns) throughout the simulation compared to the starting confirmation (unphosphorylated WT at time 0 ns) (Figure 5). S137 had more substantial fluctuations of all residues (more prominent in resides 50 to 120, especially in the range 49 to 94) than WT and T189 (Figure 5A). This range of amino acid residues contains residues His-51 and Asp-75 and Ser-135 to form the catalytic triad. A closer look for RMSF in residues (49–94) showed an apparent increase in RMSF value in S137 residues (40–50) compared to WT and T189 (Figure 5B). S137 residues (566–586) showed a pattern close to WT and T189 (Figure 5C).

### 3.4. Hydrophobicity of the NS3–NS5 Contact Site Increases with Phosphorylation

**Figure 6:**
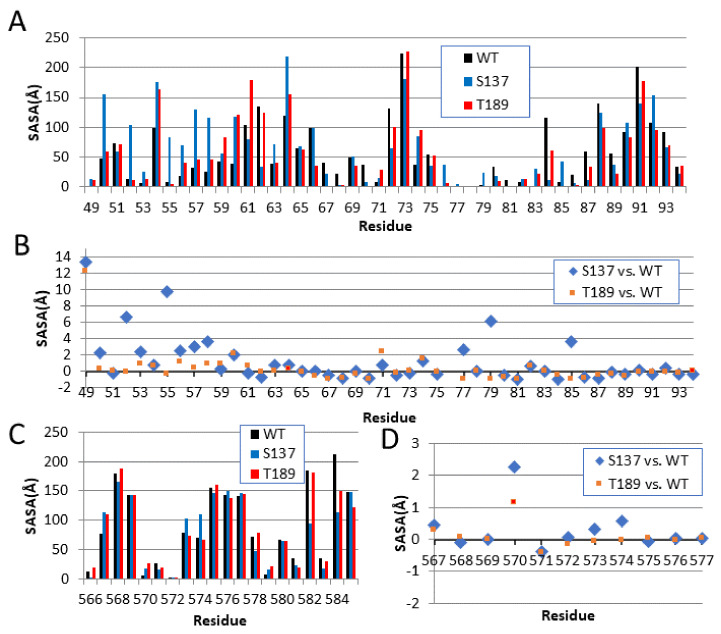
The solvent accessible surface area (SASA) of the three “100 ns” MD simulated structures (unphosphorylated WT at time 100 ns, S137, and T189 at time 100 ns). Phosphorylation of S137 increases the solvent accessibility at many of the residues. (**A**) SASA scores for residues 49–95 of WT (Black), S137 (Blue), and T189 (Red) at 100 ns simulation time, (**B**) The difference in SASA score for residues 49–95 (S137 vs. WT in Blue) and (T189 vs. WT in Red) normalized with respect to WT value. (**C**) SASA scores for residues 566–585 at 100 ns simulation time, WT (Black), S137 (blue), and T189 (Red). (**D**) The normalized difference in SASA score for residues 566–585 (S137 vs. WT in Blue) and (T189 vs. WT in Red).

Computing solvent accessible surface area (SASA) is one of the widely accepted methods to measure the changes in the accessibility of protein to solvent. SASA typically accounts for the free surface area of water molecules within a radius of 1.4 Å. In this paper, The SASA of MD simulated structures (WT, S137, and T189 during the simulation time 100 ns) was calculated. The higher the SASA score, the more hydrophilic the residue, suggesting that the atoms are exposed more to water. This SASA may indicate an interaction site with other proteins, such as NS5 or a protein kinase. Figure 6A shows the SASA score for residues 49–95 for the WT (Black), S137 (Blue), and T189 (Red) at simulation time 100 ns. Figure 6B shows the differences in the SASA scores between S137 and WT (blue) and T189 and WT (red) normalized with respect to the WT score. The differences between S137 and WT are larger than the differences between T189 and WT, most notably between residues 50–58 and 77–85. Figure 6C shows the SASA scores for residues 566–585. Unlike S137, the SASA score differences between S137 and WT compared to the SASA differences between T189 and WT for residues 566–585 are very small, if any (Figure 6D). These results are consistent with the MD trajectories RMSD and RMSF, consistent with earlier work showing changes of the catalytic site for NS3 (49–95) and less change to the helicase domain [44].

### 3.5. Solvent Accessibility in the NS3-NS2B Interaction and the Catalytic Domain

**Figure 7:**
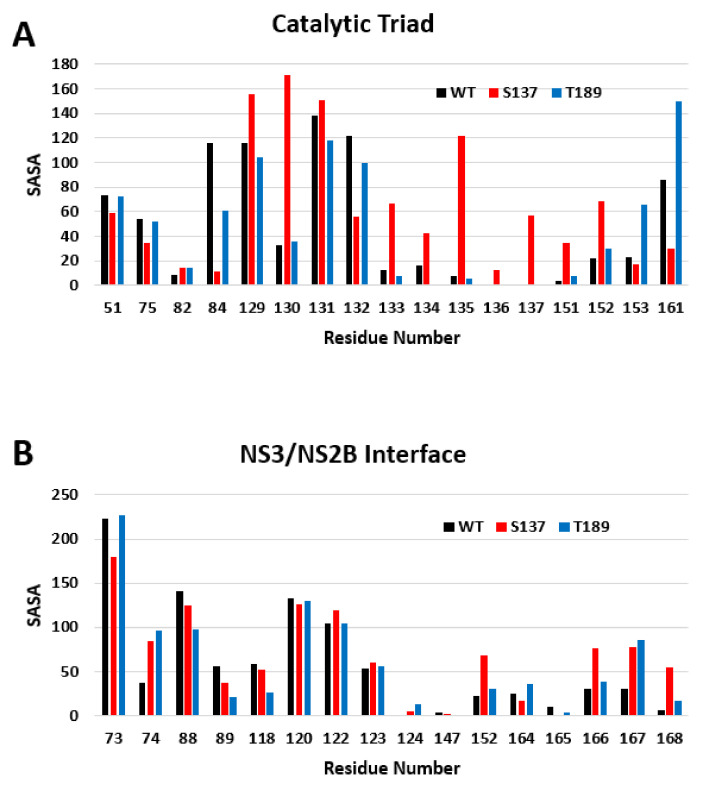
SASA changes of functional domains with phosphorylation. (**A**) The catalytic domain of NS3 shows significant changes with S137 phosphorylation (WT (Black), S137 (Blue), and T189 (Red)). (**B**) The NS3/NS2B interface has only minor changes with phosphorylation.

The N-terminus residues 1–169 are considered to be the protease domain. Previous studies highlight a peptide bound to NS3 and show that residues 51, 82, 84, 129, 130, 131, 132, 133, 134, 135, 136, 137, 151, 152, 153, and 161 are involved in the catalytic domain and the binding of the peptide Bx-NKRR-H [59]. The SASA scores for these residues are very different in the protein phosphorylated at S137 compared to the unphosphorylated form (WT) and the protein phosphorylated at T189 (Figure 7A). In fact, the size of the catalytic domain pocket was calculated using CASTp nearly doubles in size with phosphorylation at S137 from 377.5 Å3 to 756.8 Å3. NS3 needs to be complexed with NS2B in order to have its protease function intact [60]. In another study, the residues at the interface of NS3/NS2B were found to be 73, 74, 88, 89, 118, 120, 122, 123, 124, 147, 152, 164, 165, 166, 167, and 168 [61]. The SASA scores of these residues remained relatively unchanged after phosphorylation as S137 (Figure 7B).

### 3.6. Phosphorylation Induced Site-Specific Structural Changes

**Figure 8:**
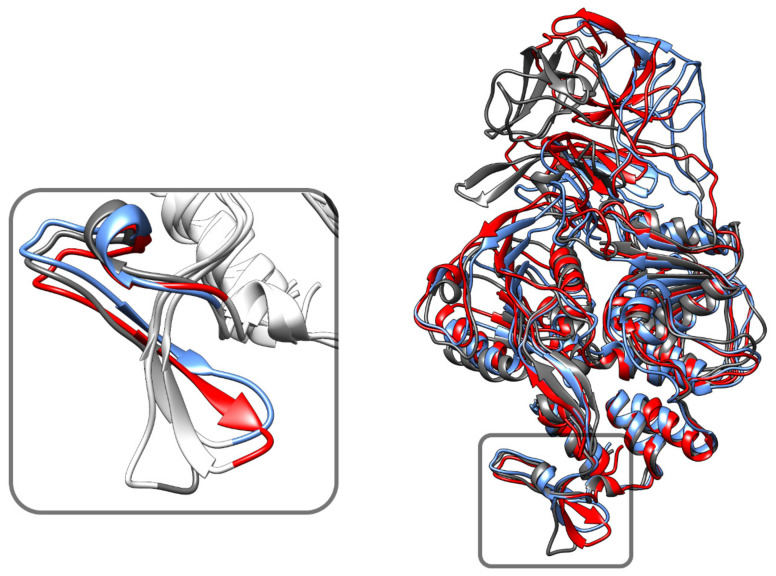
Site-specific changes in NS3 structure with phosphorylation. The C-terminal amino acid residues are essential for the interaction of NS3 with NS5. The three simulated structures at time 100 ns (gray—WT, red—S137, blue—T189). WT residues 566–585 (inside the box) show an apparent shape and position difference from S137.

Detection of increased negative-strand RNA synthesis by real-time RT-PCR for the NS3 N570A mutant suggests that NS3–NS5 interaction plays a vital role in the balanced synthesis of positive negative-strand RNA for robust viral replication [62]. In particular, the 50 C-terminal amino acid residues are essential for this interaction. After 100 ns of simulation, the phosphorylation effect shows a shape and positional difference of WT residues (566–585) from the same residues in S137. (Figure 8). The shape of WT and T189 are similar although there is a positional shift. This is consistent with the simulation results shown in Figure 5. This raises the possibility that the phosphorylation of residues S137 in NS3 might disrupt the NS3–NS5 interaction through changes in the structure at residues 566–585.

### 3.7. Docking Analysis of NS3–NS5 Interaction

We used ClusPro2 (protein-protein blind docking tool) To assess the NS3 specific-site phosphorylation on the NS3–NS5 macromolecule stability based on the docking energy. Lower energy indicates a more stable interaction. We docked three simulated structures (unphosphorylated WT at time 100 ns, T189 at time 100 ns, and S137 at time 100 ns) against the NS5. ClusPro uses the following equation to calculate the binding energy:

*E* = 0.40*Erep* + −0.40*Eatt* + 600*Eelec* + 1.00*EDARS* (1)

**Figure A3:**
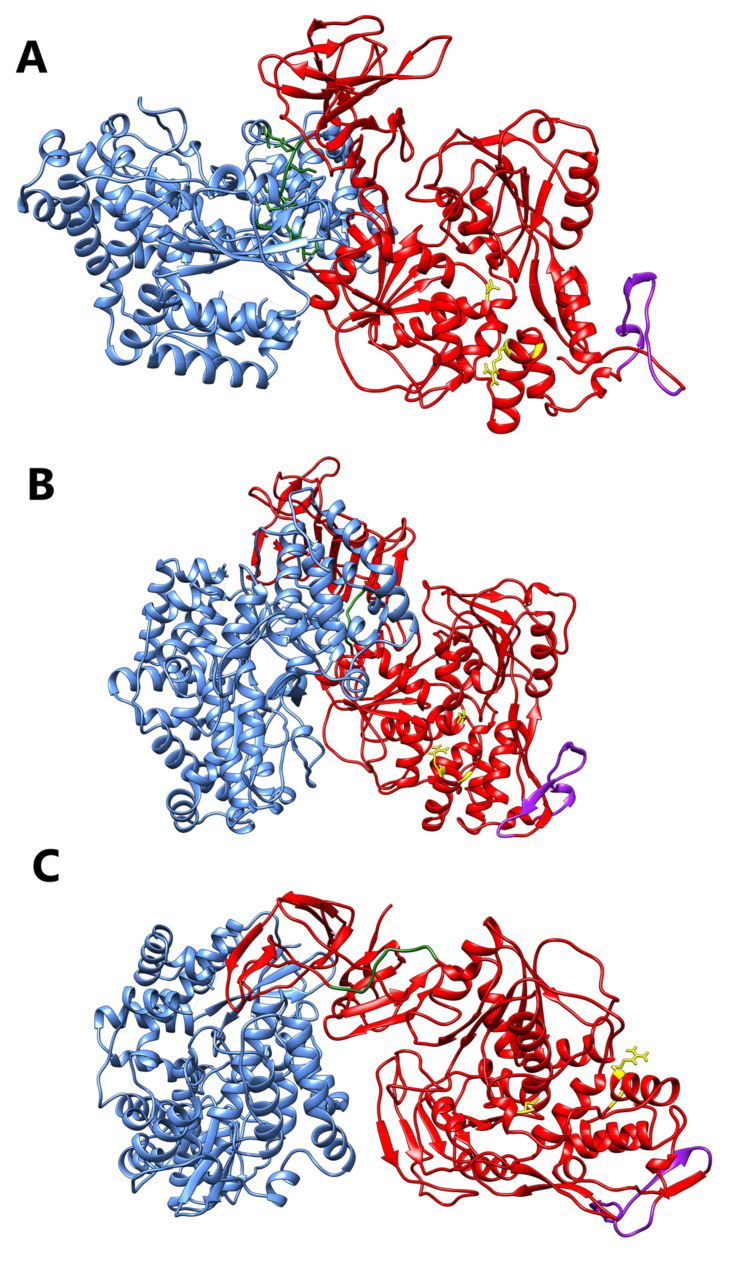
Docking of NS3 (red) to NS5 (blue). (**A**) wild type (WT), (**B**) phosphorylated at S137, (**C**) phosphorylated at T189. The NS3 residues 566–585 are shown in purple. The linker region residues 169–179 ae shown in green. The residues shown in yellow are residue numbers S137, A164, and Q167 to allow comparison of the structures.

ClusPro2 returned the 10 top poses of the interaction between NS3 and NS5 for each of the three simulated structures. In none of these were residues 566–585 were involved in the NS3:NS5 interface. As a guide to which structures were reasonable, the proposed NS3:NS5 interaction structures and information from the literature informed the choice of chosen structures [63,64]. The docking structures of NS3 and NS5 are shown in Figure A3 in Appendix A. The S137-NS5 complex had the lowest docking energy (−1141.8 kcal/mol) compared to both T189-NS5 (−949.2 kcal/mol) and WT-NS5 (−963.8 kcal/mol) (Table 6). Phosphorylation at T189 decreased the binding affinity to NS5 compared to WT. The docking results may indicate that NS3 serine 137 (S137) phosphorylation stabilizes the NS3–NS5 complex.

### 3.8. Interactions of NS3 with Quercetin

Quercetin is a plant flavonoid that experimentally inhibits Dengue replication and binds to NS3. The flavonoid Quercetin has been reported to significantly reduce dengue DENV serotype two levels by 67% [65]. Studies have measured Quercetin’s binding kinetics to NS3, estimating a K_D_ of 20 µM [14]. Computational studies using molecular docking strongly suggested Quercetin’s binding site to NS2B-NS3; however, the mechanism of viral inhibition is still unknown [13,14].

**Figure 9:**
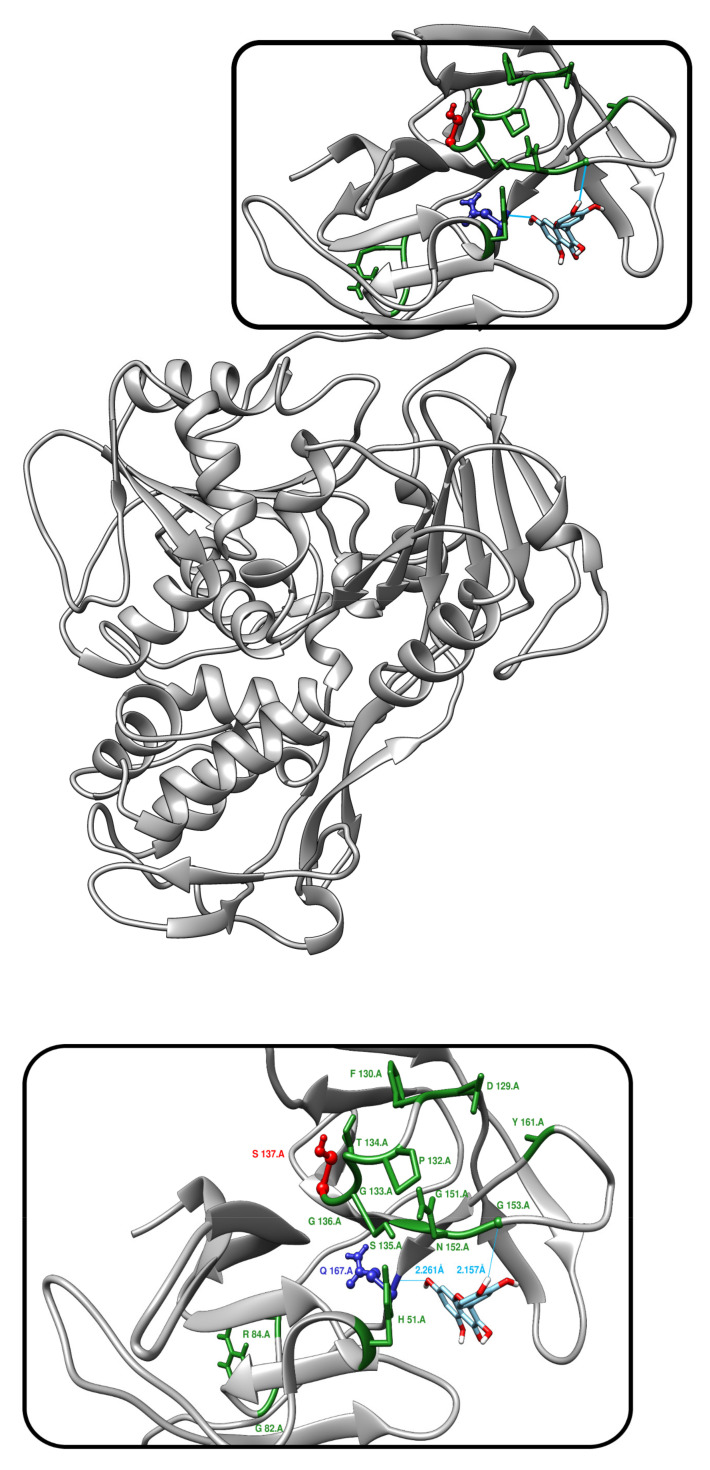
Docking of Quercetin with NS3. Auto-dock of Quercetin against the protease part of the Dengue virus shows that Quercetin binds very close to the S137 site occluding its access. S137 is shown red. Q167 is shown in blue and shares an H-bond with Quercetin. The catalytic triad residues are shown in green. The other H-bond is shared with G153.

Auto-dock Vina yielded 10 poses of Quercetin against the protease part of the Dengue virus NS3 Protease-Helicase. The site of docking is similar to those found in previous studies (13, 14). The binding free energy of these poses ranges from −6.4 to −7.4 kcal/mol. AutoDock Vina calculations showed a binding affinity of −6.4 kcal/mol for Quercetin to the wild-type NS3, −6.3 kcal/mol for NS3 phosphorylated at T189 and −5.1 kcal/mol for NS3 phosphorylated at S137. This slightly lower binding affinity for the NS3 protein phosphorylated at S137 probably has limited effect. The pose with the lowest energy is shown in Figure 9. Hydrogen bonds have been added to Dengue virus NS3 Protease-Helicase before and after Quercetin docking. Two hydrogen bonds connect Quercetin to the protease part of the Dengue virus, one bond with GLN 167 and THR 166.

## 4. Discussion

The disruption of NS3–NS5 interaction results in the transport of NS5 to the nucleus, which decreases viral replication, suggesting that S137 phosphorylation aids in dengue (and flavivirus replication) [11]. Among the MAPKs, there are the JNK and p38 kinases, whose pathways are activated during DENV infection in macrophages. Previous experimental studies have found that phosphorylation by JNK, ERK1/2, and P38 MAPK is activated during Dengue infection and that the inhibitors of JNK and p38 pathways reduce the viral activity [66,67].

Analyses using GPS 3.0, NetPhos 3.1, and Scansite 3 revealed many potential candidate phosphorylation sites on NS3 for several kinases. Candidate sites were screened based on conservation across Dengue virus sequences, based on previous studies that reported that phosphoproteins are subject to more conservation in evolution than their non-phosphorylated proteins [55,56]. This combined in silico prediction ranked S137 and T189 as the top two candidates for phosphorylated sites. Phosphorylation at NS3 S137 was predicted for MAPK, GSK3, CDK1, and JNK2. Experimental studies have shown that activation of JNK enhances Dengue infection, supporting this prediction [66,67].

In our study, we explore how phosphorylation of NS3 affects the NS3–NS5 complex stability by performing docking studies after structures were obtained using molecular dynamics simulation. The two most conserved phosphorylation sites, based on computational prediction, are NS3 serine 137 (S137) and NS3 threonine 189 (T189). Docking studies show that NS3 phosphorylation at S137 increased the binding affinity between NS3 and NS5 to a much greater extent than NS3 phosphorylation at T189. In our simulations, the interface involves residues in the linker regions, as well as both the protease (C-lobe) and helicase (N-lobe) domains. The interaction with the N-lobe might explain why phosphorylation as S137 affects the NS3:NS5 binding energy. In fact, other studies have suggested that NS5 interacts with both the helicase and protease domains [57,63].

Other studies have suggested that the NS3:NS5 interaction only involves the helicase domain (C-lobe) and specifically mention residues 566–585 [62,63,68]. The observed changes in the C-terminal amino acid residues 180–618 are small. The changes in the residues 566–585, which is at the protein periphery, are only slightly larger. In the structures from the literature, there are significant differences in the proposed complex for NS3:NS5 [63,64]. In one case, the RNA threads through the RNA interaction sites in NS3 and then through NS5. In another proposed structure as the RNA unwinds, a strand interacts with NS3 and another with NS5. We do not address this issue here as it still remains an open question. All the published docking models of NS3 with NS5 indicate that residues 566–585 are not at the binding interface Tay and co-workers observed that peptides consisting of amino acid residues 566–585 and 566–571 reduces NS3-NS5 binding [62]. In fact, Terramoto and co-workers suggest that residues near V226, D290, and T317 are involved in the NS3–NS5 binding interface [69]. This might suggest that this peptide interacts with the binding interface. However, the peptide fragment might be different in structure than the 566–585 in the native peptide. Hence, there is no direct evidence in the paper to this segment being at the docking interface. The two published docking models of NS3–NS5 agree with our model that 566–585 does not lie in the interface between NS3 and NS5. Instead, one of these models explicitly shows that the positioning of the RNA is closed to residues 566–585 [63,64].

In the simulations here, the NS3–NS5 interaction differs for WT, S137, and T189. There is also a change in docking energy of NS3 with NS5. In our simulations, and the proposed structure of Brand and co-workers, the residues 566–585 are not at the interface [63]. Instead, it seems that these residues might instead affect the helicase activity of NS3. The deviations in the 566–585 region seem to start with a kink of varying degrees at residue N570 which has been shown in experiments to be critical to the NS3–NS5 interaction and viral replication [62].

Molecular dynamics simulations show that NS3 phosphorylation at S137 leads to a significant conformational change in the NS3 catalytic domain (N-lobe) at residues 49–94 compared to unphosphorylated NS3 WT and NS3 phosphorylated at T189. This is shown by the RMSD calculation, the RMSF, and SASA scores from the simulations. These residues are of interest because they include the catalytic domain and key residues in the catalytic triad which consists of His-51, Asp-75, and Ser-135. Further exploration of the other resides that make up the binding pocket of the protease domain are shown in Figure 7A. There are substantial changes in SASA of these residues making ligand access to the site easier.

The NS3 domain containing S137 is a crucial functionally conserved region in the flavivirus family [24]. Protein homology models have suggested that this residue is involved in the “catalytic triad” (His-Asp-Ser135) that is conserved in all flaviviruses and is necessary for viral replication [24]. Site-directed mutagenesis of NS3, which converting Ser to and Ala at residue 135 in yellow fever virus (equivalent to S137 in Dengue virus), abolished the viral replication by the NS2B-NS3 complex [70]. Computational studies have explored the catalytic triad as a drug-target in screens for potential substrates [3,71,72,73,74]. However, these studies could not link a potential drug candidate to an experimentally observed reduction in viral replication [71,72,73,74]. The proximity of NS3 S137 to this critical triad site suggests that its phosphorylation is likely to be functionally important. Our work is consistent with earlier work on the active site (catalytic triad) for NS3, which showed high mobility during MD simulation [62]. Furthermore, a mutational study shows that L75A, I77A, and I79A mutants demonstrated inefficient autoproteolysis [75]. In our simulations, the SASA scores for these residues change significantly with phosphorylation as S137, as shown in Table 7.

The S137 site seems to be buried in the PDB structure (2VBC). The low SASA score of the WT at the end of the molecular simulation reflects this. The score for the protein phosphorylated at site S137 is significantly higher. A high SASA score indicates that the residue is easily accessible, and, therefore, its phosphorylation might have a very minimal effect on the NS3 folding. On the other hand, a low SASA score informs that the residue is hardly accessible, and inhibition of its phosphorylation might have a higher impact on the protein folding and, consequently, on the NS3 interactions—with other proteins—that are essential for the viral growth and replication [76]. Experiments using mass spectroscopy have shown that phosphorylation sites are commonly buried in the protein. The mechanism of phosphorylation is that there is a recognition sequence to which the kinase binds [77]. The kinase will then phosphorylate sites close to this binding site. In the process, it is likely that the interaction of the kinase with the target protein causes a conformational change in the protein that allows for the kinase to access the previously buried phosphorylation site [78]. There are many examples of buried phosphorylation sites [76]. In fact, Somavarapu et al., 2014 showed that a significant number of phosphorylation sites identified by mass spectroscopy were located in highly inaccessible regions as measured by the SASA score [79]. They suggest that this might be possible due to conformational changes during phosphorylation. In fact, phosphorylation at S137 increases the SASA measured in our simulations from 0.37 to 57.4. The phosphorylation prediction software NetPhos 3.1 considers 3-dimensional structure when making its predictions. GPS 3.0 and ScanSite 3 only take into account the 1-dimensional sequence [80]. A newer version of GPS 5.0 released in 2020 considers, the 3D structure [81].

Previous studies have observed that the catalytic site is only partially open and, therefore, requires some structural dynamics to allow substrate binding [82]. Docking studies and crystal structures only include the binding of a small hydrophilic peptide that might not reflect the true nature of binding to this pocket [59,83,84]. There are 9 important polypeptide residues at the cleavage site [83]. Current binding models and structures do not account for how all these residues interact with NS3. The current structures provide little room for the interaction with the cleft nor for how the 9-residue linker region attached to two proteins in the polyprotein can fit into the small cleft in the crystal structure [85]. The enlargement of the cleft shown in the phosphorylated simulation might provide a structure that could accommodate this. Moreover, the dengue RNA is translated to a 335 amino acid residues polyprotein and cleaved by the NS3-NS2B protease at cleavage site that contain conserved hydrophobic residues [82,86,87]. Therefore, interaction with hydrophobic residues in NS3 is likely. Other studies have shown that beyond the hydrophilic residues of the catalytic triad, there are several hydrophobic residues that are essential to ligand binding to NS3 and its subsequent cleavage by NS3 [61,88]. Phosphorylation at S137, in essence, opens the site making it possible for the protease to act on the large polyprotein with key interactions with the hydrophobic cluster in the site, with the hydrophobic residues of the cleavage site. SASA shows slight increase in binding pocket. Interestingly, the access to the NS3 key hydrophobic cluster L115, F116, V126, A160, Y161 has also increased. Although the size of the binding pocket has increased, the accessibility to this pocket has increased greatly. This is indicated by the increased pocket size calculation that includes both the binding pocket and the accessibility path.

Several plant flavonoids have been shown to display antiviral activity against viruses in the flavivirus family, including the Dengue virus. These include Quercetin, Agathisflavone, and Myricetin, which have been shown to bind NS3 at the same site [13,14]. Quercetin binds with the highest affinity with a disassociation constant of 20 µM. Docking simulations performed in the current study predict that Quercetin binds NS3 to occlude access to the S137 phosphorylation site. Blocking the catalytic site of the protease activity (catalytic triad) can either inhibit the phosphorylation as S137 or the binding of a ligand to the catalytic site or both. Docking simulations show that Quercetin binds with similar affinity to unphosphorylated NS3 (WT), NS3 phosphorylated at T189 and the form phosphorylated as S137.

Quercetin-mediated JNK inhibition is used in treating cardiovascular diseases related to vascular smooth muscle cells (VSMC) growth and apoptosis [89]. In essence, this provides a molecular target for Dengue virus inhibition and suggests that these flavonoids are all acting at this site. The in silico findings presented here set up future experimental studies to explore this hypothesis, advance the current understanding of Dengue infection, and may provide ways to inhibit viral replication. Future studies will also include experimental verification that flavonoids such as Quercetin act at this site to prevent phosphorylation at residue S137. Quercetin is predicted to bind this site with low affinity to be a suitable drug. Therefore, other compounds that bind this site would need to be developed. Such studies would be followed by optimizing lead compounds, possibly similar to Quercetin, Agathisflavone, and Myricetin, since having the molecular target and structure would allow a pharmacophore model to be developed.

## 5. Conclusions

To our knowledge, this is the first study that examined the structural effects of NS3 specific amino acid residue phosphorylation on protein structure and its impact on NS3 and NS5 interaction, and consequently, on DENV viral replication. In summary, the computational analysis and molecular simulations presented in the study make four predictions. First, we predict that the phosphorylation of NS3 at S137 strengthens its association with NS5. Second, these studies predict that JNK phosphorylates NS3 as the S137 site. Third, the study predicts that Quercetin and other plant flavonoids inhibit viral replication by binding near this site to obstruct access to S137. Fourth, given the high degree of homology of this region of NS3 in the flavivirus family, this presents a common potential mechanism across members in this family, except perhaps Hepatitis C virus, which lacks S137 (Figure A2 and Figure A3). Although these predictions are consistent with existing experimental studies, future work is needed to test these hypotheses.

## Figures and Tables

**Table 1:** ANCOVA of the simulated structures RMSD (all residues) after adjusting for the time steps.

Effect	DFn	DFd	F	ges	*p*
Time Step	4795	9590	4.443	0.69	<0.0001
Category	2	9590	59671.861	0.926	<0.0001

DFn = degrees of freedom in the numerator, DFd = degrees of freedom in the denominator, F = the F-distribution (F-test), ges = is the generalized effect size, *p* = statistical significance level.

**Table 2:** Estimated marginal means (emmean) pairwise comparisons of the simulated structures RMSD (all residues).

				RMSD Emmean Comparisons ^1^		
Category	Emmean	SE	DF	S137	T189	WT
S137	7.785	0.012	14385	NA	<0.0001	<0.0001
T189	3.871	0.012	14385	<0.0001	NA	<0.0001
WT	5.396	0.012	14385	<0.0001	<0.0001	NA

^1^ Bonferroni adjusted *p* values. Emmean = Estimated Marginal Means, SE = Standard Error, DF = Degrees of Freedom.

**Table 3:** ANCOVA of the simulated structures RMSD (residues 49–94) after adjusting for the time steps.

Effect	DFn	DFd	F	ges	*p*
Time Step	4795	9590	0.843	0.297	1
Category	2	9590	1296396.55	0.996	<0.0001

DFn = degrees of freedom in the numerator, DFd = degrees of freedom in the denominator, F = the F-distribution (F-test), ges *=* is the generalized effect size.

**Table 4:** RMSD difference from WT of structural and functional domains.

ID	N-Lobe Catalytic Domain 120–1168	Linker 169–179	NS3 Loop 566–585	C-Lobe Helicase 180–618
WT-S137	28.97 Å	8.82 Å	3.66 Å	2.82 Å
WT-T189	16.20 Å	9.78 Å	3.38 Å	3.05 Å

**Table 5:** Estimated marginal means (emmean) pairwise comparisons of the simulated structures RMSD (residues 49–94).

				RMSD Emmean Comparisons ^1^		
Category	Emmean	SE	DF	S137	T189	WT
S137	11.002	0.0045	14385	NA	<0.0001	<0.0001
T189	1.908	0.0045	14385	<0.0001	NA	<0.0001
WT	1.733	0.0045	14385	<0.0001	<0.0001	NA

^1^ Bonferroni adjusted *p*.values. Emmean = Estimated Marginal Means, SE = standard error, DF = degrees of freedom.

**Table 6:** NS3–NS5 docking energies.

ID	Cluster Members	Weighted Score Lowest Energy ^a^
WT-NS5	29	−963.8 Kcal/mol
T189-NS5	28	−949.2 Kcal/mol
S137-NS5	49	−1141.8 Kcal/mol

^a^ ClusPro docking results (protein-protein blind docking). The balanced form of the equation has been used. Cluster members = the number of structures in the cluster, Weighted Score Lowest Energy = the lowest energy found in the cluster.

**Table 7:** Solvent accessible surface area (SASA) score for residues 75, 177, and 179.

Residue	WT	S137	T189
75	54.42	35.07	52.11
177	100.35	22.45	122.17
179	56.54	100.64	82.61

## Data Availability

Data will be deposited into the Mason Archival Repository Service (MARS) at https://hdl.handle.net/1920/11628 (accessed on 13 October 2021) upon manuscript acceptance.

## Supplementary Materials

The following are available online at https://hdl.handle.net/1920/11628, Supplementary Materials in the Mason Archival Repository Service (MARS repository at https://hdl.handle.net/1920/11628 (accessed on 13 October 2021)).

## Appendix A

**Table A1:** The top predicted phosphorylation sites.

GPS			Netphos			ScanSite		
Kinase	Position	Score	Kinase	Position	Score	Kinase	Position	Score
*MAPK*	*9*	*23.1*	*cdk5*	*9*	*0.59*	*CDK5*	*9*	*0.64*
SRC	23	10.1	SRC	23	0.49			
PIKK	34	9.57						
PKC	45	9.56				PRKCZ(PKC)	45	0.52
			PKC	122	0.55	PRKCE(PKC)	68	0.49
PIKK	127	9.45						
*MAPK*	*131*	*28*	*cdk5*	*131*	*0.68*	*GSK3A*	*131*	*0.56*
MAPK	134	11.3				MAPK3	135	0.61
***MAPK***	***137***	***28.8***	***GSK3***	***137***	***0.49***	***CDK1***	***137***	***0.6***
			PKC	163	0.53			
			PKC	168	0.68			
						PRKDC(PIKK)	171	0.58
***AKT***	***189***	***10.9***	***PKB(AKT)***	***189***	***0.73***	***CAMK2G***	***189***	***0.53***
			PKC	200	0.73			
			PKC	218	0.71			
*MAPK*	*244*	*25.7*	*cdk5*	*244*	*0.65*	*CDK1*	*244*	*0.63*
			PKC	266	0.85			
*MAPK*	*271*	*31.4*	*cdk5*	*271*	*0.65*	*AKT1*	*271*	*0.69*
			PKC	293	0.53	PRKCD(PKC)	293	0.49
			PKC	301	0.67			
*MAPK*	*317*	*28.3*	*cdk5*	*317*	*0.65*	*MAPK3*	*317*	*0.63*
*MAPK*	*317*	*28.3*	*cdk5*	*317*	*0.65*	*MAPK3*	*317*	
			PKC	352	0.6	GSK3A	321	
			PKC	358	0.63	CAMK2G	358	
PKC	364	9.68				PRKCA(PKC)	364	
			PKC	386	0.61	CAMK2G	386	
PKC	389	10.7				PRKACG(PKC)	389	
PKC	453	9.95						
SRC	472	11.9						
MAPK	500	29.9				MAPK3	500	
PKC	507	9.59						
SRC	523	10.3						
						AKT1	602	
*MAPK*	*317*	*28.3*	*cdk5*	*317*	*0.65*	*MAPK3*	*317*	
			PKC	352	0.6	GSK3A	321	
			PKC	358	0.63	CAMK2G	358	
PKC	364	9.68				PRKCA(PKC)	364	0.53
			PKC	386	0.61	CAMK2G	386	0.56
PKC	389	10.7				PRKACG(PKC)	389	0.6
PKC	453	9.95						
SRC	472	11.9						
MAPK	500	29.9				MAPK3	500	0.59
PKC	507	9.59						
SRC	523	10.3						
						AKT1	602	0.71
PKC	364	9.68				PRKCA(PKC)	364	0.53
			PKC	386	0.61	CAMK2G	386	0.56
PKC	389	10.7				PRKACG(PKC)	389	0.6

Top 19 phosphorylation site candidates for each phosphorylation prediction software tool. Sites identified by all three software are shown in italics. The SASA scores for the 5 sites identified by the 3 methods are: S137-0.003; T189-0.073; T244-0.197; S271-0.183; T317-0.031. The two most highly conserved sites are shown in bold.
